# Stereotactic Radiosurgery and Fractionated Stereotactic Radiotherapy With a Linear Accelerator (LINAC) for Acromegaly Remission: Clinical Experience From a Tertiary Neurological Center in Latin America

**DOI:** 10.7759/cureus.88708

**Published:** 2025-07-24

**Authors:** Rosa Iris Santos-Santos, José Guillermo Flores-Vázquez, Luis Alberto Rodriguez-Hernandez, Irving Fuentes-Calvo, Ivan Abdiel Rodríguez-Hernández, Edgardo de Jesús Mateo Nouel, Eliezer Villanueva-Castro, Marco Antonio Muñuzuri-Camacho, Ricardo A. Palacios-Rodríguez, Xavier Wong-Achi, Rodolfo Villalobos-Díaz, Tomas Moncada-Habib, Sergio Moreno-Jiménez, Guillermo Axayacalt Gutierrez-Aceves, Lesly A Portocarrero-Ortiz

**Affiliations:** 1 Departmment of Internal Medicine and Endocrinology, Clinica Unión Médica, Santiago, DOM; 2 Department of Neuroendocrinology, National Institute of Neurology and Neurosurgery “Manuel Velasco Suárez”, Mexico City, MEX; 3 Department of Neurosurgery, National Institute of Neurology and Neurosurgery “Manuel Velasco Suárez”, Mexico City, MEX; 4 Department of Neurosurgery, National Institute of Neurology and Neurosurgery "Manuel Velasco Suárez", Mexico City, MEX

**Keywords:** acromegaly, linac, pituitary neoplasms, radiotherapy, stereotactic radiosurgery

## Abstract

Background and objective

Fractionated stereotactic radiotherapy (FSRT) and stereotactic radiosurgery (SRS) are commonly used in patients with growth hormone (GH)-secreting pituitary adenomas (PAs) who are not candidates for surgery, have residual disease postoperatively, or have failed or cannot access medical therapy. It is also considered a first-line option in elderly patients or those with comorbidities that contraindicate surgery. In this study, we aimed to evaluate the long-term outcomes of SRS and FSRT in patients with acromegaly who remained biochemically active despite prior surgical and/or medical treatment.

Methods

This was an observational, analytical, longitudinal, and retrospective study with an 11-year follow-up. Patients were divided into two groups according to the radiotherapy modality (SRS or FSRT, both delivered via a linear accelerator (LINAC)). Outcome measures included biochemical remission, hypopituitarism, and visual function.

Results

A total of 140 patients were included; 105 patients received SRS and 35 received FSRT between 2004 and 2015. Among SRS-treated patients, 41.11% (n=43) achieved biochemical remission at three years. In the FSRT group, no complete biochemical remissions were achieved at six years of follow-up.

Conclusions

Stereotactic radiotherapy is an effective adjuvant treatment for acromegaly. SRS demonstrated progressive biochemical remission with acceptable long-term toxicity. FSRT remains a therapeutic alternative when SRS is not feasible. These findings, from a Latin American public neurosurgical center, endorse the feasibility of implementing stereotactic techniques in resource-limited settings.

## Introduction

Pituitary adenomas (PAs) are the most common tumors affecting the pituitary gland, accounting for approximately 90% of all sellar lesions and 10-15% of all primary central nervous system (CNS) tumors. Based on clinicopathological characteristics, PAs are classified into the following subtypes: somatotroph, lactotroph, thyrotroph, corticotroph, and gonadotroph adenomas [1,2]. In line with recent developments in tumor pathology, the 2022 WHO Classification of Endocrine and Neuroendocrine Tumors has redefined these neoplasms under the term pituitary neuroendocrine tumors (PitNETs), reflecting their origin from adenohypophyseal neuroendocrine cells. This change acknowledges the potential for invasive behavior, recurrence, and even metastasis seen in some tumors-features not compatible with the traditionally benign implications of the term “adenoma.” By aligning pituitary tumors with the broader category of neuroendocrine neoplasms, this classification facilitates more accurate diagnosis and therapeutic planning across endocrine tumor types [3].

A cornerstone of this updated classification is the cell lineage determination based on transcription factor expression. Rather than relying exclusively on hormone production, PitNETs are now classified according to the lineage of their cell of origin, identified by key transcription factors: PIT1 (for somatotroph, lactotroph, and thyrotroph tumors), TPIT (corticotroph tumors), and SF1 (gonadotroph tumors). Ancillary markers like ERα and GATA3 assist in further refinement. This biologically informed approach improves the accuracy of subtype identification and provides better prognostic information, particularly relevant in functional tumors such as those causing acromegaly [3].

Among all PAs, 10-15% are somatotroph adenomas, predominantly functional and secrete excess growth hormone (GH). These tumors are the leading cause of gigantism and acromegaly, conditions characterized by elevated levels of GH and insulin-like growth factor 1 (IGF-1), depending on whether epiphyseal closure has occurred [4,5]. Timely diagnosis and multidisciplinary management of these tumors remain challenging. Surgery is considered the first-line treatment, followed by medical therapy, particularly in patients who are not surgical candidates or who exhibit persistent disease postoperatively. Somatostatin analogues (SSAs) are regarded as the first-line pharmacological agents in such cases [6,7].

Radiotherapy plays a crucial role in the treatment of residual or recurrent disease, especially when tumor invasion into the cavernous sinuses limits the effectiveness of surgical resection. It is also considered a primary treatment option in elderly patients or those with contraindications to surgery or SSAs. Conventional radiotherapy has demonstrated hormonal and tumor control rates of approximately 80-94%; however, stereotactic radiosurgery (SRS) offers the advantages of reduced morbidity, minimized radiation exposure to surrounding brain tissue, and improved long-term outcomes [8].

SRS was initially developed to treat intracranial neoplasms that were either inaccessible or associated with high surgical risk, and to minimize unnecessary complications linked to open surgery or chemotherapy. Its development required the collaboration of specialists in physics, radiology, engineering, and computer science. Today, SRS is a well-established treatment modality for intracranial tumors that are not amenable to conventional surgical approaches. While radiation therapy remains a cornerstone for the control and potential cure of certain brain neoplasms, precise calculation of both the radiation dose and the targeted area is essential to avoid neurotoxicity and optimize patient outcomes [9].

Currently, there is a lack of data regarding the outcomes of SRS and stereotactic radiation therapy (SRT) in the management of acromegaly within our population. Therefore, evaluating the long-term benefits of these modalities in achieving structural and biochemical control in affected patients is of particular interest, which prompted us to undertake this study.

## Materials and methods

We conducted an observational, analytical, longitudinal, and retrospective study including all patients with a confirmed diagnosis of acromegaly treated at the Endocrinology Department of the National Institute of Neurology and Neurosurgery "Manuel Velasco Suárez" in Mexico City, Mexico, between 2004 and 2015. Data were obtained through a comprehensive review of both physical and electronic medical records. 

Inclusion criteria consisted of patients aged 18 years or older with a biochemical diagnosis of acromegaly, defined by elevated GH levels and an IGF-1 index >1.2. Patients had to have received treatment with either SRS or fractionated stereotactic radiotherapy (FSRT), with or without prior surgical intervention (microscopic transsphenoidal, endoscopic transsphenoidal, or transcranial approaches). Patients with a clinical follow-up of less than six months, those with incomplete biochemical data that precluded assessment of remission, or those who received treatment with conventional (non-stereotactic) radiotherapy modalities were excluded.

Biochemical remission was defined as a nadir GH level <1 ng/mL following an oral glucose tolerance test (OGTT) and/or an IGF-1 index <1.2. Treatment response was assessed by serial measurements of nadir GH and IGF-1 index at six months post-radiotherapy and annually thereafter, for up to six years. Additionally, the functional status of the thyrotropic, corticotropic, and gonadotropic axes was evaluated periodically, along with visual field assessments using automated perimetry.

## Results

A total of 140 patients were included, all of whom received some form of ionizing radiation as either primary or adjuvant therapy. Two main treatment groups were identified: 72% (n=105) received SRS, while 28% (n=35) underwent FSRT. Of the total cohort, 60% (n=84) were female and 40% (n=56) were male, with a median age of 37 years (16-60 years). Surgery was the initial therapeutic approach in 86.4% (n=121) of patients. Surgical modalities included microscopic and endoscopic transsphenoidal approaches, as well as transcranial surgery. Specifically, 70% (n=98) underwent a microscopic transsphenoidal approach, 10% (n=14) an endoscopic transsphenoidal approach, and 6% (n=9) were treated via transcranial surgery. 

Patients with persistent active disease following surgery were treated with FSRT until 2008, after which SRS became the preferred radiotherapeutic modality. Visual field assessment was performed at baseline in 97% (n=137) of patients. Of these, 72% (n=101) showed no visual field impairment. Of the 26% (n=36) with visual field deficits, 13% (n=18) exhibited superior temporal field loss, 4% (n=5) had amaurosis, and 2% (n=3) presented with bitemporal hemianopia. 

Stereotactic radiosurgery (SRS) 

A total of 105 patients received SRS. Of these, 87.6% (n=92) had undergone prior surgical treatment. The most commonly used surgical approach was the microscopic transsphenoidal technique, performed in 84% (n=78) of these cases. Patients with persistent active disease following surgery were subsequently treated with SRS. The mean baseline levels of nadir GH and IGF-1 before SRS were 5.63 ng/mL (range: 0.10-39.8) and 540.5 ng/mL (range: 0.87-1147), respectively. The mean IGF-1 index was 1.3 (range: 0.3-3.25). The mean prescription dose of SRS was 22 Gy (range: 16.6-32.2). 

No cases of biochemical remission were observed during the first six months following SRS. At one year, 10% (n=10) of patients achieved remission, defined as nadir GH <1 ng/mL. At 24 and 72 months, remission was observed in 17.9% (n=18) and 38.4% (n=40) of patients, respectively. At five years, 42% (n=44) had achieved biochemical remission. Regarding the IGF-1 index, 9.3% (n=9) of patients had an index <1.2 at one year. This proportion increased to 17.9% (n=18) at two years and reached 41.1% (n=43) at three years, reflecting a favorable progressive trend in disease control over time. 

Thyrotropic axis function was evaluated in 57.1% (n=60) of patients. At baseline, 27% (n=16) presented with hypothyroidism. Among the 73% (n=44) who were initially euthyroid, 9.1% (n=4) developed de novo hypothyroidism at six months post-SRS. This figure rose to 29.4% (n=12) at three years, 33.3% (n=14) at four years, and peaked at 44% (n=19) at five years of follow-up. Corticotropic axis evaluation was available for 55.2% (n=58) of patients. Baseline hypocortisolism was observed in 12.1% (n=7). Among the 87.9% (n=51) with normal function at baseline, 11.7% (n=6) developed hypocortisolism at six months, increasing to 51.3% (n=26) by year three. 

**Figure 1:**
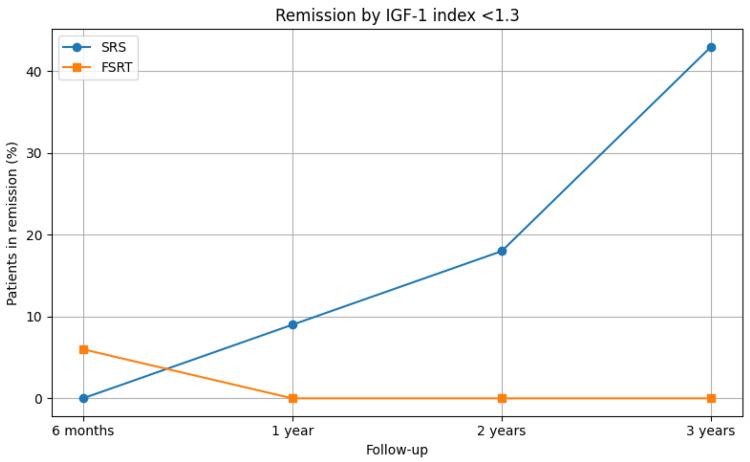
Remission rates by IGF-1 index <1.3 in patients treated with SRS or FSRT Percentage of patients achieving biochemical remission based on IGF-1 index <1.3 over time following SRS or FSRT. Progressive remission was observed only in the SRS group, with no sustained remission in the FSRT group after the first six months FSRT: fractionated stereotactic radiotherapy; IGF-1: insulin-like growth factor 1; SRS: stereotactic radiosurgery

Gonadal axis evaluation was performed in 52.3% (n=55) of patients. At baseline, 41.8% (n=23) had hypogonadism. Among the 58.2% (n=32) with normal gonadal function, 25.0% (n=8) developed hypogonadism at six months, increasing to 47.4% (n=15) at four years (Figure 1). Regarding visual field status, 98.1% (n=103) of patients underwent baseline assessment. Of these, 18.4% (n=19) presented with visual field defects. At six months post-SRS, 15.7% (n=13) of previously unaffected patients developed new deficits, while 13.7% (n=2) of those initially affected showed improvement. The majority (70.6%, n=36) remained stable, with no further changes observed during the six-year follow-up period. 

Fractionated stereotactic radiotherapy (FSRT)

A total of 35 patients were treated with FSRT. Of these, 65.7% (n=23) were female and 34.3% (n=12) were male, with a mean age of 37 years (range: 15-59 years). Previous pituitary surgery had been performed in 82.9% (n=29) of patients. Among these, 68.9% (n=20) underwent a microscopic transsphenoidal approach, 10.3% (n=3) an endoscopic transsphenoidal approach, and 20% (n=6) a transcranial approach. Patients with persistent active disease following surgery were treated with FSRT. The mean radiation dose administered was 55.5 Gy (range: 13.8-91.5). 

Regarding biochemical remission, 83.3% (n=29) of patients continued to exhibit elevated nadir GH levels (>1 ng/mL) at six months post-treatment. Additionally, 85.7% (n=30) maintained an elevated IGF-1 index. No cases of complete biochemical remission were observed at four, five, or six years of follow-up. Thyrotropic axis function was assessed in 62.9% (n=22) of patients. At baseline, 40.9% (n=9) had hypothyroidism. Among the initial euthyroid patients, 7.7% (n=1), 23.5% (n=5), 37.5% (n=8), 42.8% (n=9), and 57.1% (n=12) developed de novo hypothyroidism at six months, two, three, four, and five years, respectively. 

Corticotropic axis evaluation was also performed in 62.9% (n=22) of patients. At baseline, 22.7% (n=5) had hypocortisolism, while 77.3% (n=17) were eucortisolemic. Among the latter, 23.5% (n=4) developed hypocortisolism at six months, peaking at 41.2% (n=7) at two years. Gonadotropic axis function was evaluated in 62.9% (n=22) of patients. At baseline, 61.9% (n=13) had hypogonadism, which remained unchanged throughout the five-year follow-up period (Figure 1). 

With respect to visual field outcomes, 97.1% (n=34) of patients underwent baseline evaluation. Of these, 50.0% (n=17) exhibited visual field defects. Among the 50.0% (n=17) who were initially unaffected, 23.8% (n=4) developed new visual deterioration at six months, while 76.2% (n=13) remained stable. Among those with baseline deficits, 11.7% (n=2) demonstrated improvement. No additional changes were observed during the six-year follow-up. 

Overall, the long-term adverse effects of both SRS and FSRT were predominantly related to progressive dysfunction of the pituitary hormonal axes. In contrast, visual outcomes tended to stabilize after the first six months, with partial recovery observed in a subset of patients. A summary of the demographics and relevant clinical characteristics of the study participants is presented in Table 1.

**Table 1:** Demographics, treatment characteristics, visual outcomes, remission, and endocrine dysfunction in SRS and FSRT patients Biochemical remission was defined as GH nadir <1 ng/mL and/or IGF-1 index <1.3. Pituitary axis dysfunction was evaluated longitudinally and reported as the cumulative number of patients with new-onset hypothyroidism, hypocortisolism, or hypogonadism during follow-up FSRT: fractionated stereotactic radiotherapy; GH: growth hormone; IGF-1: insulin-like growth factor 1; SRS: stereotactic radiosurgery; VF: visual fields

Variables	SRS	FSRT
Total patients, n	105	35
Male	44	12
Female	61	23
Median age, years	37.81	37
Previous surgery	92	29
Microscopic transsphenoidal	78	20
Endoscopic transsphenoidal	11	3
Transcranial	3	6
No surgery	13	6
Mean dosis, Gy (range)	22 (16.6-32.2)	55.5 (13.8-91.5)
Basal VF evaluation, n	103	34
No VF affection	84	17
VF affection	19	17
Bitemporal hemianopia	0	3
Unilateral hemianopia	2	2
Amaurosis	3	2
Cuadrantanopia	1	2
Superior temporal VF affection	13	5
VF after 6 months of radiation		
New VF affection	13	4
Improvement from previous affection	2	2
Stable VF	36	13
Remission by GH nadir <1 ng/mL		
6 months	0	6
1 year	10	0
2 years	18	0
3 years	40	0
Remission by IGF-1 index <1.3		
6 months	0	6
1 year	9	0
2 years	18	0
3 years	43	0
Thyrotropic axis evaluation	60	22
Baseline hypothyroidism	16	9
Baseline euthyroidism	44	13
Hypothyroidism at 6 months	4	1
Hypothyroidism at 1 year	6	2
Hypothyroidism at 2 years	9	5
Hypothyroidism at 3 years	12	8
Hypothyroidism at 4 years	14	9
Hypothyroidism at 5 years	19	12
Corticotropic axis evaluation	58	22
Baseline hypocortisolism	7	5
Baseline eucortisolism	51	17
Hypocortisolism at 6 months	6	4
Hypocortisolism at 1 year	14	4
Hypocortisolism at 2 years	17	7
Hypocortisolism at 3 years	26	7
Gonadotropic axis evaluation	55	22
Baseline hypogonadism	23	13
Baseline eugonadism	32	9
Hypogonadism at 6 months	8	0
Hypogonadism at 1 year	9	0
Hypogonadism at 2 years	9	0
Hypogonadism at 4 years	15	0

**Figure 2:**
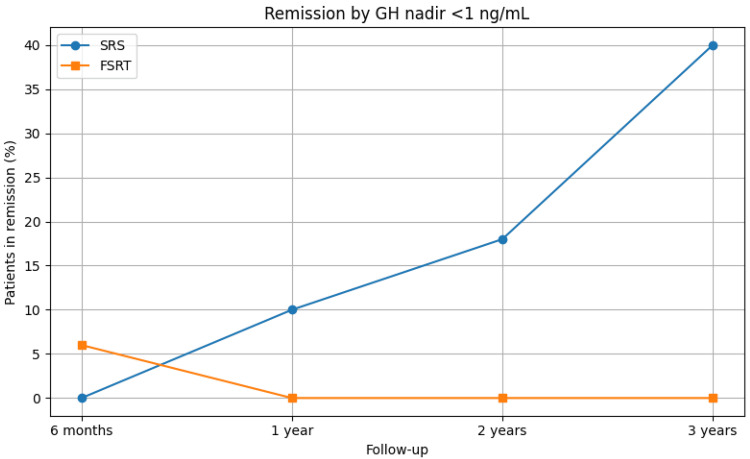
Remission rates by GH nadir <1 ng/mL in patients treated with SRS or FSRT Percentage of patients achieving biochemical remission defined by GH nadir <1 ng/mL during follow-up after SRS or FSRT. SRS demonstrated a progressive increase in remission rates, whereas FSRT showed no cases of sustained remission after the first year FSRT: fractionated stereotactic radiotherapy; GH: growth hormone; SRS: stereotactic radiosurgery

**Figure 3:**
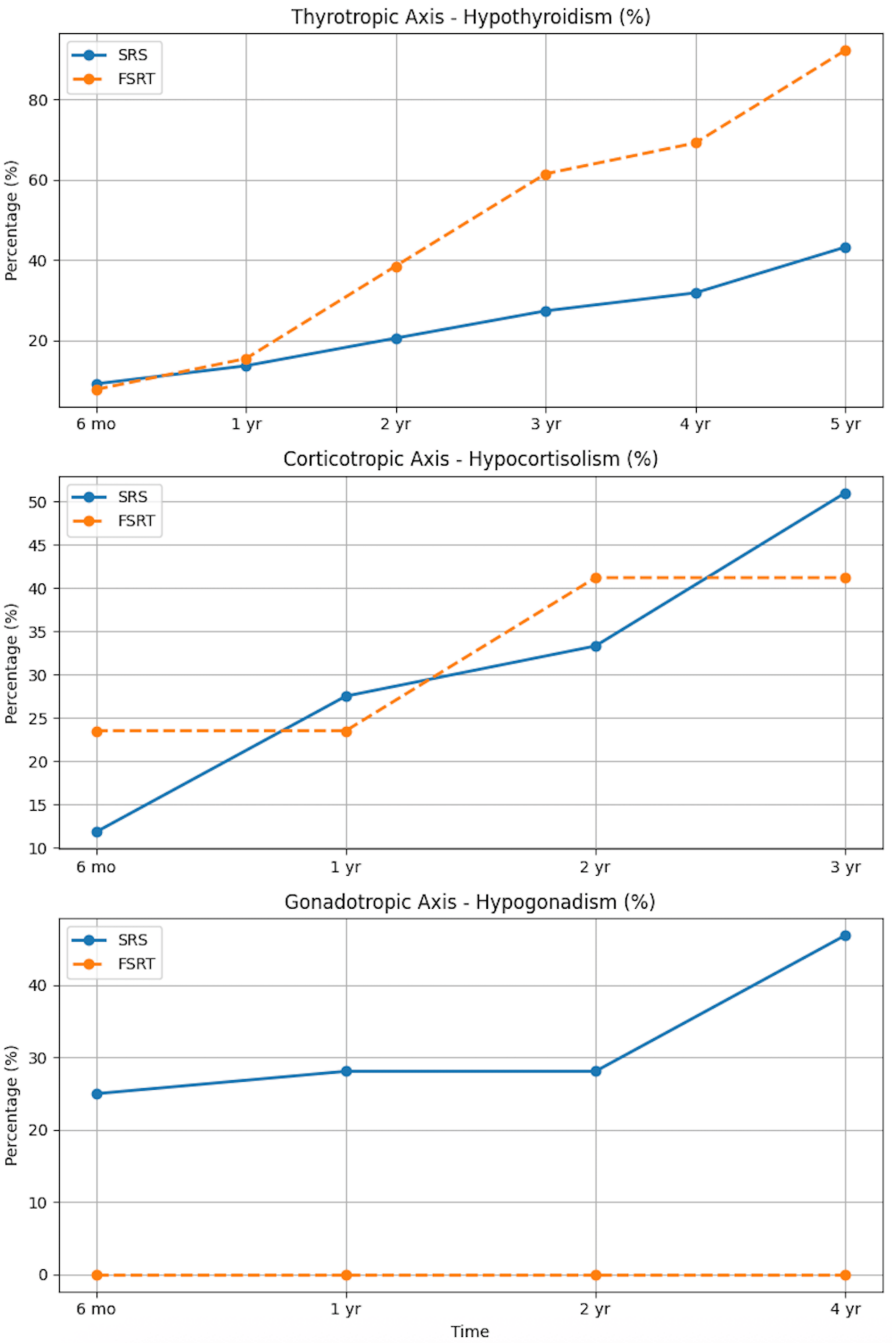
Longitudinal progression of hypopituitarism by axis after SRS and FSRT Percentage of patients developing hypothyroidism, hypocortisolism, or hypogonadism during follow-up after SRS or FSRT. Top: thyrotropic axis. Middle: corticotropic axis. Bottom: conadotropic axis FSRT: fractionated stereotactic radiotherapy; SRS: stereotactic radiosurgery

Figure 2 illustrates the remission rates by GH nadir <1 ng/mL in patients treated with SRS or FSRT, while Figure 3 depicts the longitudinal progression of hypopituitarism by axis in patients following SRS and FSRT.

## Discussion

Ionizing radiation therapy, in its various modalities such as SRS and FSRT, has gained prominence as an adjuvant treatment for acromegaly. It serves as a viable alternative for patients who are not suitable candidates for surgery, exhibit poor biochemical control after surgery, or present with residual tumor. Nevertheless, its application has traditionally been limited by long-term adverse effects, most notably hypopituitarism in approximately 50% of cases and visual impairment in up to 15%. As a result, radiotherapy is generally reserved for patients in whom both first- and second-line treatment options have been exhausted [10]. Advanced techniques such as SRS and FSRT have been developed to minimize radiation exposure to surrounding healthy tissues and to enable more precise, localized dose delivery [11]. Several studies have evaluated biochemical control and long-term toxicity associated with these modalities, although with varying follow-up durations. 

This study involved the largest national cohort to date to evaluate the use of SRS and FSRT for the treatment of acromegaly in a tertiary referral center. The main objective was to assess disease remission, pituitary axis function, and visual field integrity. Our data show that SRS led to progressive biochemical remission in up to 42% of patients at five years, while no remissions were observed in the FSRT group during long-term follow-up. Both treatment modalities were associated with progressive dysfunction of the pituitary hormonal axes, while visual outcomes remained largely stable after the first six months.

The remission rate achieved with SRS in our study (42% at five years) is consistent with previous reports, which range from 35% to 60%, depending on follow-up duration, marginal dose, and baseline disease burden. Jiun-Lin et al. [12] evaluated 22 patients with residual or recurrent functional pituitary adenomas between 1994 and 2004, all of whom received LINAC-based SRS and were monitored for at least three years. Residual or recurrent disease was defined by persistently elevated GH or IGF-1 levels and imaging-confirmed tumor presence after prior surgery. Biochemical remission, defined as fasting GH <2.5 ng/mL and age- and sex-adjusted normal IGF-1 levels, was achieved in 68.2% of patients. Post-radiotherapy global hypopituitarism persisted in 22.7% of cases. 

In contrast, the lack of remission observed in our FSRT group diverges from some published series reporting variable remission rates (10-80%), particularly with higher total doses and longer follow-up durations. Cheng-Chia et al. [13] reported a 76% remission rate at six years in a cohort of 176 patients treated with radiotherapy, using criteria based on GH and IGF-1 index. Their findings suggest that earlier initiation of radiotherapy may be associated with higher remission rates. Similarly, Singh et al. [14] demonstrated that IGF-1 normalization improved progressively over time, increasing from 23-42% at five years to 45-50% at ten years post-radiotherapy, with cumulative doses of 45-50 Gy delivered in 25-28 fractions. These outcomes were associated with improvements in systemic complications and overall quality of life. Yang et al. [15] in a large cohort study of 970 patients with acromegaly, reported an overall disease control rate of approximately 48% post-radiotherapy, highlighting the progressive enhancement of outcomes through advances in radiotherapy planning. 

Our finding of delayed remission after SRS supports the hypothesis of a time-dependent cytotoxic effect of high-dose focused radiation on somatotroph adenomas. The progressive increase in IGF-1 index normalization over three years aligns with previous studies. However, further data regarding the radiological features of the pituitary tumors are needed to explore whether the endocrine response truly lags behind radiological or anatomical changes. 

With regard to endocrine toxicity, both SRS and FSRT were associated with significant dysfunction over time, particularly affecting the corticotropic and thyrotropic axes. In our study, any form of hypopituitarism was observed in up to 50% of patients treated with ionizing radiation. When evaluating individual pituitary axes, similar rates of dysfunction were found between SRS and FSRT. Thyrotropic deficiency occurred in 44% of patients treated with SRS versus 57% with FSRT. Corticotropic deficiency was present in 51% of SRS cases compared to 41% in the FSRT group. Gonadotropic dysfunction rates were 47.4% for SRS and 0% for FSRT. These findings are consistent with previous reports, which show hypopituitarism in 30-60% of patients within five years. Knappe et al. [15] reported a significantly lower risk of adrenocorticotropic deficiency after SRS compared to conventional radiotherapy (HR: 0.54; 95% CI: 0.30-1.00; p=0.049). It was further noted that no deficiencies in sex or thyroid hormones were observed when the mean pituitary dose remained below 15 Gy, and cortisol deficiency was also absent under these conditions [16]. Our findings reinforce the need for long-term hormonal surveillance in this patient population. 

Visual outcomes remained largely stable, with a small proportion of patients even showing improvement. These findings are consistent with those of Singh et al., who reported a correlation between visual toxicity and higher marginal prescription doses. The estimated incidence of visual toxicity ranged from 1.2% to 7.4% for marginal doses between 20 and 35 Gy [16]. 

Among the strengths of our study are the size of the cohort, the prolonged follow-up (up to six years), and the fact that the data reflect real-world clinical practice in a public neurosurgical referral center in Latin America, within the context of a developing healthcare system. However, this study has several limitations, including its retrospective design, incomplete follow-up in a subset of patients, absence of radiological data, and lack of uniform biochemical assays throughout the study period. Additionally, the two treatment groups were not contemporaneous and may reflect changes in institutional protocols over time. 

Our results suggest that SRS is a viable and effective option for patients with persistent or recurrent acromegaly after surgery, achieving progressive remission in a significant subset. FSRT may remain a useful alternative when SRS is not feasible; however, close and prolonged monitoring of endocrine function is essential in both treatment groups. 

## Conclusions

Stereotactic radiotherapy, particularly SRS, is an effective adjuvant treatment for acromegaly, achieving progressive biochemical remission with acceptable long-term toxicity. While FSRT remains a viable alternative when SRS is not feasible, both modalities require close endocrine follow-up. Our findings, gathered from a Latin American public neurosurgical center, highlight the feasibility and relevance of these techniques in resource-limited healthcare settings.
